# Polyelectrolytes: From Seminal Works to the Influence of the Charge Sequence

**DOI:** 10.3390/polym15234593

**Published:** 2023-11-30

**Authors:** Nam-Kyung Lee, Min-Kyung Chae, Youngkyun Jung, Albert Johner, Jean-Francois Joanny

**Affiliations:** 1 Department of Physics and Astronomy, Sejong University, Seoul 05006, Republic of Korea; lee@sejong.ac.kr; 2 National Institute for Mathematical Sciences, Daejeon 34047, Republic of Korea; mkc@nims.re.kr; 3 Supercomputing Center, Korea Institute of Science and Technology Information, Daejeon 34141, Republic of Korea; yjung@kisti.re.kr; 4 Institut Charles Sadron CNRS-Unistra, 6 rue Boussingault, 67083 Strasbourg, France; 5 Institut Curie, Physique des cellules et Cancer, Collège de France Soft Matter and Biophysics Chair, 11, PSL University, Place Marcelin-Berthelot, 75231 Paris, France; jean-francois.joanny@college-de-france.fr

**Keywords:** polyelectrolytes, polyampholytes

## Abstract

We propose a selected tour of the physics of polyelectrolytes (PE) following the line initiated by de Gennes and coworkers in their seminal 1976 paper. The early works which used uniform charge distributions along the PE backbone achieved tremendous progress and set most milestones in the field. Recently, the focus has shifted to the role of the charge sequence. Revisited topics include PE complexation and polyampholytes (PA). We develop the example of a random PE in poor solvent forming pearl-necklace structures. It is shown that the pearls typically adopt very asymmetric mass and charge distributions. Individual sequences do not necessarily reflect the ensemble statistics and a rich variety of behaviors emerges (specially for PA). Pearl necklaces are dynamic structures and switch between various types of pearl-necklace structures, as described for both PE and PA.

## 1. Introduction

Modern polymer science started with the 1920 paper by Staudinger [1], who introduced the concept of long molecules comprising many repeating units held together by ordinary covalent chemical bonds. A nice account of Staudinger’s manifesto (which has never been translated) was given in 2020 for its 100th anniversary [2]. The emergence of the concept of covalent polymer is contemporary to the early development of quantum mechanics. Considering the scale of science, it is a young field. Staudinger himself was inspired by natural rubber and later also studied biopolymers, foreshadowing the extended scope of the field as it stands. Polymer science including polymer chemistry, polymer physics, biological aspects and applications grew to an extent which precludes the project of an exhaustive review.

Water, which may still be termed abundant, is an ecologically harmless solvent, in contrast to most organic solvents. Unfortunately, polymer backbones are typically insoluble in water. Some polymer backbones, which can establish hydrogen bonds, are nonetheless soluble in water, among which the most popular are Polyethylene Oxide (PEO) [3] and Poly(N-isopropylacrylamide) PNIPAM. Various theoretical descriptions for uncharged water-soluble polymers were proposed: de Gennes suggested that the Flory–Huggins type theory with three body attractions can lead to closed-loop solubility diagrams [4], and detailed lattice (Flory–Huggins type) theories were developed [5,6], which generated induced three-body attractions [6]; water-soluble polymers were later described as annealed hydrated/dehydrated copolymers [7]. The solubility of these polymers in water decreases with increasing temperature (the loop diagram is usually not closed), in contrast to most polymers in organic solvents. PNIPAM changes solubility in water at about body temperature and attracted considerable academic attention [8,9]. PEO is also of considerable practical interest [10].

**Figure 1:**
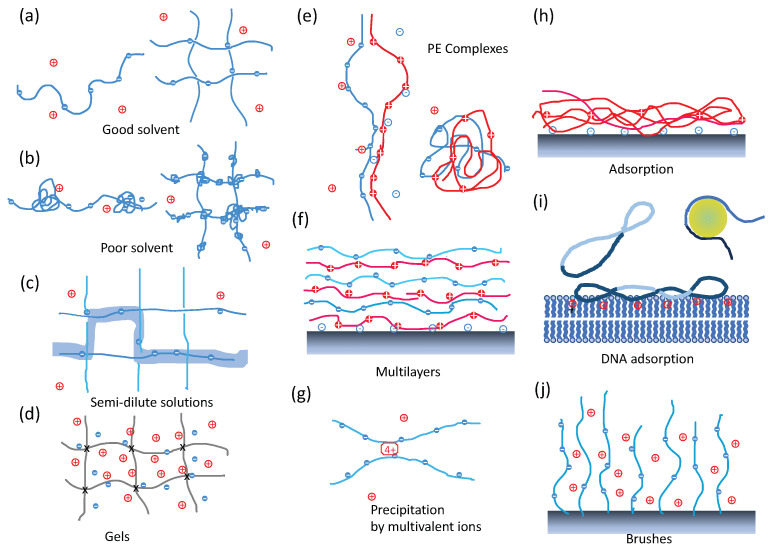
(**a**–**c**) PE solutions. (**a**) Left—Single PE chain in good solvent which is stretched by electrostatics with a few counterions. Right—PE solution in good solvent where the correlation length corresponding to typical distances between chains is represented as the mesh size, without physical crosslinks. (**b**) Left—Overall stretched pearl-necklace structure of a single PE chain in poor solvent. Right—PE solution in poor solvent where meshes interconnected by pearls appear. (**c**) Semi-dilute solution: The depicted polyelectrolyte configuration (2D illustration) gains entropy by selecting orientations at each ‘crossing’. In the case of an intrinsically flexible polyelectrolyte, the correlation length, illustrated as the distance between chains, can be qualitatively equated to the electrostatic persistence length. The thick shadow line represents a conformation of a test chain. (**d**) PE gel. Crosses (×) indicate permanent (chemical) crosslinks. The osmotic pressure of trapped counterions tends to swell the gel. (**e**) PE Complexes. Left—Ladder structure with an open loop. Right—Scrambled-egg structure. (**f**) Multilayers. Sketch of a periodic multilayer structure. (**g**) PE precipitation mediated by multivalent ions. (**h**,**i**) PE adsorption on a substrate. (**h**) Intrinsically flexible PE. (**i**) rigid PE. Left—DNA plasmid on a planar substrate. Right—ds-DNA on a colloid/histone. (**j**) PE brushes. PEs are grafted to a solid substrate by one end and form a so-called brush. The brush of flexible PEs is swollen by the osmotic pressure of the trapped counterions.

Besides these important exceptions, most water-soluble polymers carry charged monomers whose charge is compensated by counterions. They are generically called polyelectrolytes (PEs) and are the topic of this paper (see Figure 1). We try to provide a consistent minimal path through the literature on PEs, allowing to embrace the field in its actual state. Many original important contributions are hence not mentioned and are replaced by subsequent works on similar topics; this partially reflects the taste of the authors.

The objective of this review is to provide the development/history of the field though essential papers that have substantially improved our understanding. Besides, we push forward the point of view that the recent trend focuses on sequence-dependent properties, bringing the field somewhat closer to biophysics, although this evolution is of considerable interest for material science, too. Generic PE properties based on a description with the charge as a continuous variable, smeared out along the polymer are presented first. Then, we proceed to more recent investigations on PE systems, where the charge sequence plays an important role. The last part describes some of our own recent works on the structure of random PE sequences in poor solvent, with a few results on random polyampholytes (PAs) for comparison. The final discussion also briefly mentions approaches and topics that deserve further attention.

From the perspective of electrical charges, we may distinguish between polymers that carry charges of both signs and polymers that carry charges of the same sign. Polymers carrying charges of both signs along their backbone are called polyampholytes (PAs) [11]. Most proteins are PAs. Proteins, unlike random synthetic PAs, generally have a rugged energy landscape and a well-defined ground state, not ruled by electrostatics only, which determines their biological activity. An important class of proteins known as Intrinsically Disordered Proteins (IDPs) [12] which does not have a well-defined ground state shares many features of synthetic PAs. This PA character of IDPs is well documented experimentally [13,14] and has attracted a lot of attention recently, including from theorists [15,16]. Continuous attention was given to the phase behavior of IDPs or proteins with disordered protein regions (IDPRs) and other associative biomacromolecules in water [17], sometimes in close relation with membrane-less organelles [18,19]. For clarity, we call polyelectrolyte (PE) a polymer carrying charges all of the same sign as opposed to PAs, from here on.

Throughout, a continuous description of water is used, which considers water as a dielectric fluid with a dielectric constant $\epsilon_{r}=80$. This is fair in the dilute and semidilute polymer solutions considered below. The contrast in dielectric constant between the polymer and the water may become relevant in the dense PE phase [20]. Because of its high dielectric constant, water serves as a good solvent for ions. Typically, it is energetically more advantageous to distribute counterions within water rather than creating a shared (ionic) structure that is locally neutralized with the polymer. The entropy of released counterions is driving the dispersion of the counterions and polymer in water. The charged polymer backbone stretches out under the influence of electrostatic repulsions. Added salt screens the electrostatic interaction, so adding salt is an easy way to tune the properties of PE solutions.

## 2. Generic Descriptions Using Smeared out Charge Distributions

Among the essential properties of single chains, two prevalent phenomena are the distribution of counterions around the chain and the stiffening of the chain due to electrostatic repulsion. The question as to which extent counterions are released by the polymer into the solution was raised early on by osmotic pressure measurements, and concepts of charge regulation and charge condensation were introduced, as discussed in the book by Oosawa [21]. This very classical question obtained an improved solution (beyond mean-field) more recently; see, for example, reference [22] and references therein. The case of strong coupling of the counterions, mainly of higher valency, to the polymer was considered in the same work [22]. The condensed counterions form a more-or-less rigid crystal-type structure on the cylinder, mimicking the rigid PE backbone.

The bending rigidity is of special importance for biopolymers such as double-stranded DNA (ds-DNA) and is typically characterized in terms of the persistence length, which qualitatively separates small scales, where the backbone behaves as stiff, and large scales where it behaves as totally flexible. The dependence of the persistence length upon added salt was well characterized for intrinsically rigid PEs like ds-DNA and understood [23]. Measurements of the bending rigidity as a function of ionic strength on other PEs were roughly split into two categories: part of the measurements were consistent with the variation observed for ds-DNA and with ref. [23], others, where the persistence is mainly due to electrostatic repulsion, were in conflict [24]. This puzzle regarding the variation in the persistence length with salt attracted considerable efforts from theory [24,25,26,27] and simulations [28]. Simulations mainly support the model of Khokhlov and Katchatourian of a rod of electrostatic blobs [29]. Quantitative theories [24,26] are of variational type. Reference [24] predicts two regimes with an unusually complex crossover. The subsequent ref. [26] explains the weakness of ref. [24] and identifies the previous Khokhlov and Katchaturian behavior, in accord with simulations. These theoretical works [24,26] and simulations [28] utilize the Debye–Hückel screened interaction potential rather than explicit salt ions. On the other hand, explicit salt ions raise questions about the very interaction between the salt ions and the PE also beyond electrostatics. The emerging picture is conceptually much simpler than that presented in ref. [24], but the question of the persistence length remains, to some extent, open. The iconic example of ds-DNA benefited from a recent advanced theory [30] which allowed us to refine the measured value of its intrinsic rigidity. The two limitations of the standard theory, namely the weak charge hypothesis and the vanishing chain thickness, are typically not fulfilled in real systems. The authors relax both limitations in a relatively simple way, leading to a compact expression for the persistence length [30].

**Figure 2:**
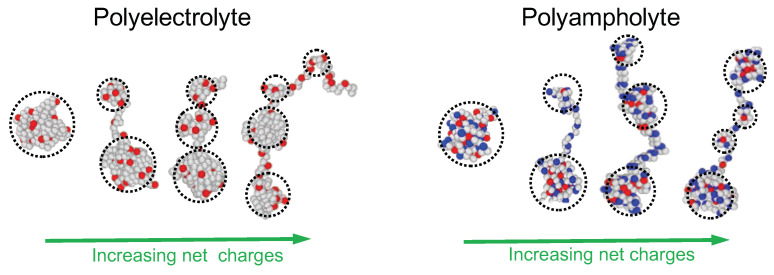
Pearl-necklace conformations of PE in poor solvent conditions (**left**) and pearl necklaces of random PA sequences in weakly poor solvent conditions (**right**). The necklace structure consists of pearls (indicated by dashed circle) and connecting strings. The number of pearls increases with increasing net charges. The conformations are obtained from MD simulations for PA and PE chains consisting of *N* = 202 monomers in which every 3rd monomers can carry charges (p=3). All charge sequences are created by unbiased Markovian processes. Blue and red colors represent different types of charges. The net charges of the chains increase from left to right: $Q_{PE}$ = 16, 22, 28 and 34 for PE chains and $Q_{PA}$ = 12, 16, 20 and 24 for PA. (See, Supplementary Materials for simulation model).

The understanding of the behavior of a single polymer chain is a good starting point for comprehending the behavior of solutions in the same solvent. This is, to some extent, true for PEs, too. The seminal paper by de Gennes, Pincus, Velasco and Brochard [31], published in 1976, describes correlations in PE solutions and gives a theoretical description of PE solutions from a physical point of view. Several important points are made in their work. It is shown that the single PE (dilute regime) is stretched with an extension proportional to the number of monomers and the electrostatic blob concept is introduced; the upper critical dimension 6 is given; and the idea of a Newton-type equation for stretched (weakly fluctuating) conformations is sketched; this was discussed later (essentially one decade later) as the classical limit of the Schroedinger-type Edwards–Lifshiz equation for the propagator [32]. It is argued that the semi-dilute solution remains isotropic (for flexible PE backbones) and one chain in the solution follows large-scale Gaussian statistics with the electrostatic persistence length equal to the correlation length. Predictions are made for the scattering function and the viscosity. The careful and inspired conclusion calls for experiments. SAXS and fluorescence emission experiments by Essafi et al. showed qualitatively different behaviors for PEs with hydrophilic and hydrophobic backbones [33]. Hydrophobic PEs do not follow predictions of ref. [31]. Earlier, Khokhlov [34] predicted the influence of the solvent quality on the structure of a dilute PE and a semi-dilute polyelectrolyte solution. The dense PE solution was subsequently described quantitatively in the framework of RPA by Borue and Erukhimovitch [35] and independently by Leibler and Joanny [36]. In ref. [34], Khokhlov introduced the cylindrical, cigar-shaped, single-PE conformation as a result of local collapse due to hydrophobicity imposing the internal density of the cylinder and global elongation under the long-range electrostatic repulsion. Although this model was recognized as unstable due to the instability of the charged cylinder demonstrated by Rayleigh [37], some fairly elongated cylindrical sections can nonetheless be found mainly in random PAs under special conditions as discussed in Section 4. The pearl-necklace conformation was introduced by Dobrynin, Rubinstein and Obukhov [38] for PEs and by Kantor and Kardar for PAs [39]. (See Figure 2.) The authors of Ref. [38] also proposed an elegant description of the semi-dilute PE solution based on the same pearl-necklace model. As a result, PEs in poor solvent (with a hydrophobic backbone) and PE in good solvent (with a hydrophilic backbone) need to be considered separately.

A series of reviews [40,41,42] marks out a period of intense (theoretical) activity roughly ending around the year 2005.

We concentrated here on progress in our fundamental understanding of PE where the single chain and solutions are key. Besides solutions (Figure 1a–c), other PE-based systems were considered: PE gels [43] (Figure 1d), complexes of polyanions and polycations [44] (Figure 1e), PE multilayers [45] (Figure 1f), precipitation or folding of PEs by multivalent counterions [46,47,48] (Figure 1g), PE adsorbed at an interface [41,49] (Figure 1h,i), and grafted PEs [50,51] (Figure 1j), to cite just a few. These systems are sketched in Figure 1 with an extended caption. Compacted PE complexes in short COPECS, sometimes also called saloplastics, obtained via (ultra)centrifugation or extrusion of well-selected PE complexes under high salt, exhibit enhanced mechanical properties and represent a promising variant of PE complexes for applications [52,53,54,55].

## 3. Charge Sequence Effects

The considerable progress in our generic understanding of PEs achieved in the seminal papers cited earlier is solely based on a smeared-out charge description, accompanied by the idea that the importance of the charge sequence is marginal due to the long-range character of the electrostatic interaction (except for the case of somewhat extreme charge correlations). In contrast, smearing out the charge of a PA does not make sense and the charge correlations are essential [56,57]. It turns out that the sequence does matter for polyanion/polycation complexation. Indeed, complexation was shown to depend on the charge correlation along the PEs: charge blockiness favors complexation [58]. A comprehensive account of recent progress on complex coacervation can be found in the recent reviews by Rumyantsev [59] and by Sing and Perry [60]. In the same spirit, the influence of the sequence on self-complexation of PAs [56,57], where charges of both signs are located on the same polymer, was re-examined recently [61,62]. A complete phase diagram was also given for IDPs [15]. As to the structure of a single PE (or PA) chain, the sequence is specially important for partially collapsed structures like pearl necklaces. Figure 2 illustrates various conformations of pearl necklaces composed of pearls connected by strings. It is energetically favorable to form strings between pearls out of overcharged subsequences which link uneven pearls, provided the smaller pearls carry a higher charge density [63,64]. In other words, it is favorable to localize a relative charge excess on average in strings than in pearls [62,64], and also in smaller pearls as compared to larger ones [65]. This is easier for annealed charges (say, pH-dependent charges) where the actual sequence can adapt to the structure, but is impossible for quenched regular sequences. How easily these inhomogeneous structures can be achieved for quenched PEs depends on the detailed charge sequence [64].

In the following section, we explore the structure of PE pearl necklaces featuring quenched charge sequences. The theoretical picture is supported by Molecular Dynamics (MD) simulations. The uncorrelated random sequences and correlated sequences, generated by unbiased Markovian processes, are explored in our previous publication [65]. Here, we primarily focus on presenting analysis on the charge sequences with positive correlations, incorporating previously undisclosed data. The results are consistent with our previous findings and are explained using the same physical approach. We invite the reader to compare the results presented below with those in Ref. [65].

## 4. Hydrophobic Polyelectrolytes: Influence of the Charge Sequence

The standard pearl-necklace model for hydrophobic PE describes regular charge sequences and can be considered as a mean-field model for random PE. However, recent research has revealed that random PEs do not tend to adopt configurations with an equal distribution of pearls, as previously suggested by mean-field theory [65]. The structure of random PEs results from the interplay between the disorder along the charge sequence and thermal fluctuations. Moderately charged random PEs exhibit very asymmetric mass distributions, as depicted in Figure 2. MD simulations and energy considerations demonstrate that moderately charged random PEs primarily concentrate their mass and charge within the pearls. (As mentioned earlier, this behavior contrasts with very blocky sequences.) Moreover, random PEs do not tend to divide into evenly sized pearls; instead, they form pearl necklaces characterized by significant asymmetry. The disorder in the sequence also disrupts the correlations between adjacent pearls to some degree, resulting in the occurrence of two large pearls in close proximity (see Figure 2).

To gain further insight, we describe the energy landscape of the *n*-pearl states, taking into account the distribution of mass and charge among the pearls. The key question is how a PE with a quenched PE charge sequence can visit energetically favorable regions.

A pearl can be considered as a drop of dense polymer phase (of almost vanishing osmotic pressure) with a surface tension associated with the polymer/water(solvent) interface. With $M_{p}$ and $Q_{p}$ the total mass and charge belonging to pearls, the retained energy *E* comprises the electrostatic self-energy of the *n*-pearls and their surface energy. At this point, the energy of strings between pearls and the interaction among pearls and among strings and pearls are neglected. It is suggested by the standard pearl-necklace model where, for the necklace to be stable at all, the self-energy of the pearls must dominate. Consistently, the order of the pearls along the sequence is not considered [65]. This simplified model helps to structure the parameter space and to provide clarity when interpreting the data clouds generated by simulations. In particular, the model predicts the location of the energy extrema and their stability, which, in turn, provide a rationale for the global distribution of data points obtained from the samples (See, also Supplementary Materials). The model is expected to be less suitable when dealing with highly blocky statistics.

The configuration with mass distribution $\left\{m_{i}\right\}$ and charge distribution $\left\{q_{i}\right\}$ among the pearls is characterized by a set of asymmetry parameters for the mass distribution $\left\{x_{i}\right\}$ and charge distribution $\left\{y_{i}\right\}$. These parameters are defined by $m_{i}/M_{p}=1/n+x_{i}$ and $q_{i}/Q_{p}=1/n+y_{i}$, where $x_{i}$ is the relative excess mass of *i*-th pearl referenced to the uniform mass distribution $m_{i}=M_{p}/n$ and $y_{i}$ is similarly defined for the charge distribution. When all pearls have equal mass and charges, $x_{i}=0,y_{i}=0$. The main physics is captured by the following expression of the energy:

(1) $$\frac{E}{E_{S}}=\sum_{i=1}^{n}{\left(1/n+x_{i}\right)}^{2/3}+\chi \sum_{i=1}^{n}\frac{{\left(1/n+y_{i}\right)}^{2}}{{\left(1/n+x_{i}\right)}^{1/3}}-1-\chi .$$

The conservation of mass and charge imposes the two constraints.

(2) $$\sum_{i=1}^{n}x_{i}=0;\;\;\;\;\sum_{i=1}^{n}y_{i}=0.$$

The single globule state is chosen as the reference state and its surface energy $E_{s}$ sets the overall energy scale, while the control parameter χ measures the ratio of the electrostatic energy to the surface energy in the uni-globular reference state. The first term on the right-hand side carries a 2/3 power and represents the surface energy of actual bead *i*, while the second term stands for its electrostatic self-energy. The subsequent additive terms are non-essential and adjust the energy of the reference state to zero.

Then, the *n*-pearl structure can be pictured in a 2(n−1) dimensional space. The uniform pearl distribution corresponds to the zero vector in which all components $\left\{x_{i}\right\},\left\{y_{i}\right\}$ are zeros. An analysis of Equation (1) reveals that, in the case of moderate charge (χ<n), the state with evenly sized pearl is not the energy minimum but instead the highest energy saddle point. The configuration with n−1 evenly large pearls complemented by one small pearl represent the next highest energy saddle point, and so forth. The absolute energy minimum is reached with only one large pearl complemented by n−1 evenly sized small pearls. Note that in the mean-field description, where the condition χ=n/2<n holds, the asymmetric structure is generally expected, except for instances with unusually small numbers of pearls due to rare fluctuations. Increasing χ while keeping *n* constant results in a flatter energy landscape. Conversely, for a fixed χ value, for a fixed sequence, a realization with fewer pearls exhibits greater fluctuations within a smoother landscape. This feature also influences kinetic properties, as demonstrated later in this section.

A comprehensive analysis of the energy described in Equation (1) can be found in reference [65]. This reference explores the energy extrema associated with a specific number of pearls across the entire range of χ values. Figure 2 in ref. [65] displays the energies of non-symmetric states for systems composed of three beads. The symmetric state has the lowest energy when χ exceeds *n* (χ>n), while the non-symmetric states cease to exist beyond a critical value $\chi_{c}$, which is somewhat larger.

For MD simulations, we consider PE and PA chains consisting of *N* = 202 monomers, where every third monomer (*p* = 3) can bear a charge. All charge sequences are generated by unbiased Markovian processes. In the case of PE, every third monomer can carry a charge (1) or remain neutral (0). Correlations between charges can be positive, negative, or absent. In uncorrelated sequences, the probability of having a charge is 1/2 for each charge site, leading to the global unbiased statistics 〈Q〉≈N/(2p) for PE. Note that there are variations in net charge from sequence to sequence. We may choose a sub-sample that retains specified net charges, differing from the global average. The global sample is generated with a prescribed correlation following a Markov process. If we select a large sub-sample with an imposed (net) charge, its statistics are well-defined, but its correlation can vary. To take an extreme example: selecting a sub-sample where all sites are charged (1) results in a single correlated block, irrespective of the initial Markov process used, although such sequences may be scarce in the global sample). Positively correlated sequences tend to favor blocks of either zero or one, while negatively correlated sequences tend to alternate between charges zero and one. Below, we consider charge sequences with positive correlations which have an average block length of 4, and uncorrelated random sequences which have an average block length of 2. The former is referred as a “blocky” sequence. Among these, sequences that satisfy specific net charge conditions are selected for further analysis.

In the case of PA, our focus is on uncorrelated PA chains where either positive (+1) or negative (−1) charges are randomly assigned with a probability of 1/2 at every third monomer. The ensemble of PA chains results in a unbiased global statistics of vanishing average charge 〈Q〉=0. There are variations in net charges from sequence to sequence. The net charge of each chain is determined by the difference between the majority charge and the minority charge. Detailed simulation descriptions can be found in the Supplementary Materials.

**Figure 3:**
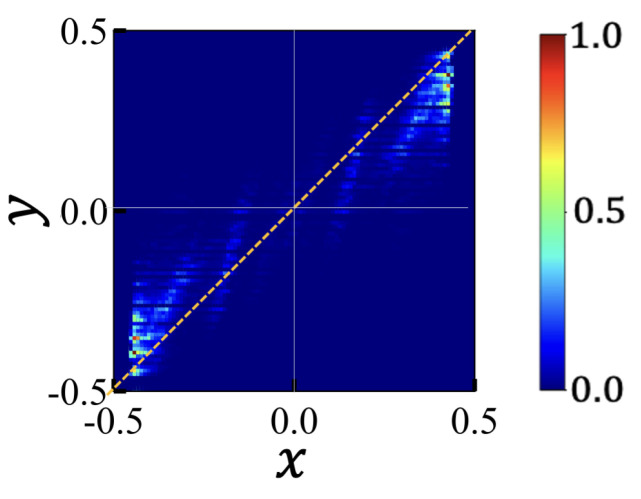
Density of states p(x,y) of a 2-pearl structure of PE chain consisting of *N* = 202 monomers and net charge *Q* = 22. Charge sequences have positive charge correlation with average block length 4. The color bar in [0,1] scale represents the density of visited states realized in MD simulations. Relative excess mass $x=m/M_{p}-1/2$ and excess charge $y=q/Q_{p}-1/2$ measure the asymmetry between the two pearls. The region where x>0 corresponds to larger pearls, i.e., $x_{1}$, while the region where x<0 corresponds to smaller pearls, i.e., $x_{2}$. The line x=y represents the uniform charge distribution where each pearl has a charge proportional to its mass. States below (above) the line indicate undercharged (overlarged) large (small) pearl states. Note that the data points exhibit point symmetry with respect to the origin, a property given by the constraints, as shown in Equation (2).

Figure 3 depicts the density of states in the space of {x,y} for PE chains when they adopt two-pearl structures. Here, ‘*x*’ and ‘*y*’ represent the relative excess mass and charge of a pearl, defined as $x=m/M_{p}-1/2$ and $y=q/Q_{p}-1/2$ for two pearl states. The line *x* = *y* corresponds to the case in which each pearl has charge in proportion to its “share” of mass (Here and below, “share” refers to the amount of charge in proportion to the mass of the pearl). For large pearls, their mass exhibits a positive excess value (x>0), and their charge distribution lies below the x=y line. This implies that the larger pearls carry fewer charges than their “share,” while the smaller pearls (x<0) carry more charges than their “share”.

**Figure 4:**
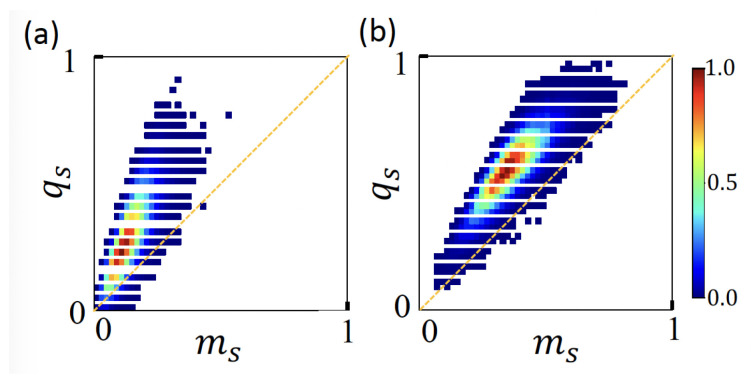
Density of states $p(m_{s},q_{s})$ of PE conformations in pearl necklaces where the number of pearls is *n* = 3. The states are evaluated according to the relative mass and charge allocated at strings, $m_{s}=N_{s}/N$ and $q_{s}=Q_{s}/Q$. Ensemble of PE chains consisting of *N* = 202 monomers and carrying net charges of (**a**) *Q* = 22 and (**b**) *Q* = 34 are considered. Each charge sequence has charge–charge correlations with the average block length of four. The color bar in [0,1] scale represents the density of visited states realized in MD simulations. With increasing net charges, more mass is allocated to the string. A greater population above the line $m_{s}=q_{s}$ indicates that strings carry more charges than what corresponds to their shares.

To better illustrate how charges are distributed between pearls and strings, we may define the reduced mass and charge at strings as $m_{s}=N_{s}/N$ and $q_{s}=Q_{s}/Q$, respectively, where $N_{s}$ and $Q_{s}$ refer total mass and charge allocated at strings. In Figure 4, we evaluate the density of states $p(m_{s},q_{s})$ of blocky PE sequences with *n* = three pearls. With increasing net charges, more mass is allocated to the string. A greater population above the line $m_{s}=q_{s}$ indicate that strings carry more charges than what corresponds to their shares.

**Figure 5:**
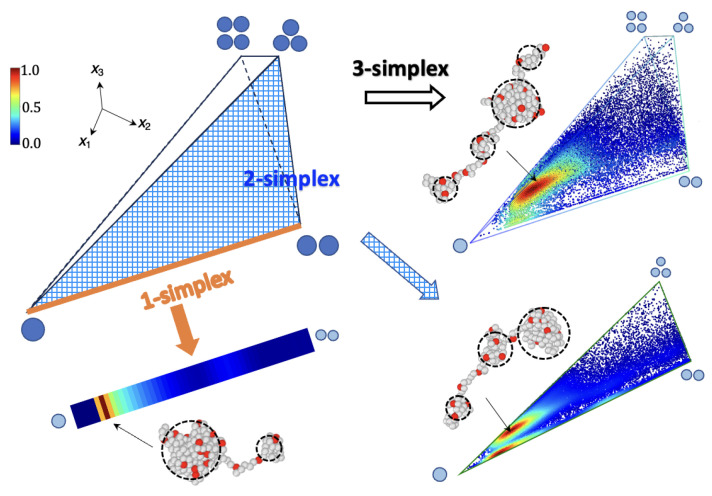
Simplex representations for PE with blocky charge sequences, *Q* = 22, *N* = 202. Mass asymmetry among *n*-pearl states are shown on (n−1) simplex representation: 1-simplex representation for two pearl states, 2-simplex representation for three pearl states, and 3-simplex representations for four pearl states. The color bar in [0,1] scale represents the density of visited states realized in MD simulations. Representative conformations are shown for each (n−1) simplex. Weakly charged PE occupies the conformational space near the vertex $V_{1,n-1}$ where one large pearl is dominant. In the presence of positive charge correlations, the asymmetry of the pearl structure is further accentuated compared to uncorrelated sequences, as demonstrated in Ref. [65]. Mass asymmetry of 4-pearl states can be shown as internal points in tetrahedron. A shaded triangular face of the tetrahedron corresponds to a 2-simplex. The thick orange line in a 2-simplex corresponds to 1-simplex. Each vertex $V_{n_{l},n_{s}}$ of tetrahedron is identified with number $n_{l}$ of large pearls (shown as blue disks).

Since we are mainly interested in the pearl size distributions, we project the parameter space to the (n−1)-dimensional sub-space spanned by the mass parameters $\left\{x_{i}\right\}$ only. After projection, the parameter space reduces to the (n−1)-simplex defined by the $x_{i}$ constraint (Equation (2)) and the ranking conditions on the pearl masses (The order of the pearls along the structure is not retained). For the *n* pearls, we exclude the smallest pearl, whose mass is set by the conservation laws but must still be positive. Additionally, there are n−1 ranking conditions of the retained masses ($x_{i}$’s). Together, these *n* linear inequalities establish the boundaries of the interior region of the (n−1)-simplex. Turning the inequalities into equalities defines the *n* sides of the (n−1)-simplex. The inequalities defining the interior of the simplex and the position of the vertices are explicitly given in the Supplementary Materials, together with equations determining the location of the energy extrema. Figure 5 shows the simplex representations of MD simulation data for two, three, and four pearls, respectively: two pearl states are represented in a one-simplex, which is a segment; three pearl states are in a two-simplex, which is a triangle; and four pearl states are shown in a three-simplex, which is a tetrahedron. Note that a face of a (n−1)-simplex is a (n−2)-simplex. In particular, the face that does not contain the origin embeds the (n−1)-pearl states. It is hence, in principle, possible (but not so appealing) to represent all states with up to *n* pearls in the (n−1)-simplex.

Each extremum of the energy Equation (1) is expressed by the number of evenly large pearls $n_{l}$ and the number of evenly small pearls $n_{s}$. We use two subscripts $n_{l}$ and $n_{s}$ to denote each energy extremum $E_{n_{l},n_{s}}$ and the corresponding vertex $V_{n_{l},n_{s}}$ (see Supplementary Materials for the coordinates of vertices and energy extrema in simplex representation). Which regions of the simplex will be more densely populated is governed by the interaction energy and the allowed topology. Two important points to note are: (1) The positions of energy extrema $E_{n_{l},n_{s}}$ coincide with the vertices $V_{n_{l},n_{s}}$ if the PE does not carry any charges. This means that in the limit of vanishing charge, the energy extrema approach is applicable to the vertices of simplex. (2) When the electrostatic self energy is moderate (χ<n), the ground state conformation at $E_{1,n-1}$ is highly asymmetric and the symmetric states $\vec{0}$ ($E_{n,0}$) are only saddle points. State $\vec{0}$ is the energy minimum only for large χ, where pearl numbers larger than χ prevail and this symmetric state is hardly observed. In the case of strongly charged polyelectrolytes (PE) where χ approaches *n*, conformations tend to concentrate significantly around the vertex $V_{n,0}$. In the case of strongly charged PE sequences where nearly all sites are charged, the charge distribution becomes nearly regular, albeit with a few defects. This effect is somewhat analogous to what is observed in poly(styrene sulfonate) (PSS), which exhibits solubility at high sulfonation rates (greater than 2/3). Charge regulation of pearls accompanied by counterion condensation on strings is expected in this limit.

When positive charge correlations are present, charge sequences tend to be blocky. In blocky sequences, the distribution of pearls remains asymmetric even when there is large net charge *Q* (note that typically, strings carry more charges than their share). The most densely populated area in a simplex does not change significantly with increasing *Q*, as compared to uncorrelated random sequences.

**Figure 6:**
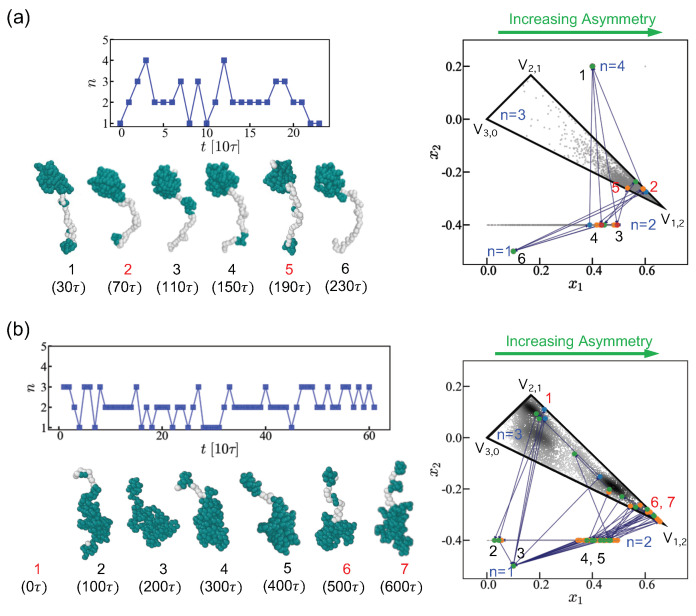
Structural changes accompanying changes in pearl number are shown for (**a**) PE and (**b**) PA. We retain the sequences from the sub-ensembles with the given net charges. (**a**) Fluctuation of PE with *Q* = 22 and *N* = 202. The conformation of pearl-necklaces is monitored with time interval 10τ and the change of the number of pearls n(t) over 230τ is shown in the upper left panel. Small pearls appear and disappear on the tail/string. Snapshots are taken every 40τ (τ is the MD time unit.). (**b**) PA pearl-necklaces with *Q* = 20 (44 majority charges and 24 minority charges) and *N* = 202. The change in the number of pearls n(t) is shown in the upper left panel over 600τ. Snapshots are taken every 100τ from MD simulations. In multiple simplexes (right panels), 1-pearl states (n=1) are represented as a point, the 2-pearl states (n=2) are depicted as a 1-simplex $x_{1}\in \left[0,0.5\right)$, 3-pearl states (n=3) are shown as triangular 2-simplex $\left\{x_{1},x_{2}\right\}$. The states with a larger number of pearls (n≥ 4) are shown as a point above the triangular 2-simplex for convenience. The chronologically numbered PE and PA forms are displayed in the corresponding simplex according to the number of pearls in the multiple simplexes. If the pearl number in the previous state is 1, 2, 3, or 4, the points on the simplex are displayed in blue, orange, green, and red, respectively. PA switches from the vicinity of V${}_{2,1}$ to the vicinity of V${}_{1,2}$ along a complex path through different number of pearls. A direct path inside the triangle (3-pearl states) is largely suppressed due to the very sparsely populated intermediate states within the triangle.

The pearl necklace is a very dynamic structure. Figure 6a shows a series of snapshots which depict fluctuation of small pearls accompanied by one large pearl. In the multi-simplex representation, the trajectory generally remains near the vertex $V_{1,n-1}$, indicating that large pearls are rather robust against thermal fluctuations. Small pearls tends to nucleate on strings, and the typical lifetime of a small pearl is about ∼10τ (with τ the MD time unit). The large pearl state with *n* = 2 has a longer lifetime of ∼50τ. Most random PE sequences, considered in a rather poor solvent, tend to have a well-defined number of large pearls. This contrasts with pearl necklaces formed by PAs in marginally poor solvent [66], where the number of large pearls also shows fast fluctuations, as depicted in Figure 6b.

The polarization energy primarily provides the cohesive part of energy for PAs. For PAs in a marginally poor solvents, the boundaries of pearls are less well defined due to the weaker surface tension. It is remarkable that a few random PA sequences can adopt multiple shapes identified by different numbers of large pearls. Figure 6b shows a series of fluctuating PA shapes with a varying number of large pearls. In the triangular 2-simplex representation, the trajectory spans both vicinities of $V_{1,2}$ and $V_{2,1}$ vertices. The time scale of these switches can be as short as ∼1000τ. The trajectories typically go through a two-pearl-state with a single large pearl where small pearls merge into and split from.

## 5. Conclusions

We tried to draw a consistent minimal route through the physics of polyelectrolytes. We privileged the tangible and intuitive route initiated by de Gennes and Pincus, which was extended in early works by Khokhlov, and we did not aim to be exhaustive. Another review could start from the Flory school of thought [67], and could include work of the Dutch group in Wageningen [68] or the numerous contributions of Muthukumar [69,70]. The development of PE physics is presented as a two-step evolution: the seminal works using charge distributions homogeneously smear out along the polymer backbone, followed by recent developments emphasizing the role of the charge sequence along the backbone.

We primarily focus on single PE chains and their behaviors in solutions. While more complex PE systems are mentioned, along with some references, a comprehensive knowledge of these systems is based on understanding single PE chains in the solution. Systems involving both polyanions and polycations, such as PE complexes, need more specific attention and are partially related to PAs.

Recently, there has been increasing emphasis on the role of charge sequences in the context of polyelectrolyte (PE) complexation, polyampholytes (PAs), and PEs in poor solvents. The statistics of the charge distribution along the backbone are typically chosen as Markovian, and it is found that blocky statistics favor complexation. In the related context of PA pearl necklaces, it is observed that as the statistics become more blocky, a higher number of charges are allocated to the strings. Those works resort to ensemble averages and to the physics of disordered systems. Chemical synthesis of PEs often shows more complex statistics in the absence of specific control. Despite this complexity, predictions regarding complexation has been successfully verified. [58].

We developed the example of random PE in poor solvent based on our own recent research. It shows that pearl necklaces are highly irregular structures showing a small number of large pearls accompanied by a majority of small pearls. In contrast to the regular sequence, interactions between pearls do not dictate the arrangement of the pearls along the necklace and neighboring large pearls occur.

Large shape fluctuations are also predicted for PAs. Individual sequences do not always reproduce the global statistics and the structure and dynamics are very sequence-dependent. This similarity to conformational plasticity, a crucial concept in Intrinsically Disordered Proteins (IDPs), is striking. While being structured in pearls, the polymer can readily adapt to external stimuli and functions as a multitasker.

One of the most relevant criterion determining properties of a given sequence is its “blockiness”. The length and number of blocks (mainly minority charge blocks when there is finite net charge) in the sequence significantly impact the behavior. Low blockiness, as also is the case for the charge sequence of IDPs, favors large shape fluctuations and labile aggregates from the identical sequences.

The control of the sequence opens the route to the control of self-complexation. Sequence specific studies seeking for sequences with peculiar properties, such as highly fluctuating structures or the control of self-aggregation of similar PAs, have indeed been conducted recently and may lead to high-tech applications. This idea is familiar to biochemists synthesizing de novo polypeptides for a specific purpose.

At the end of this study, we are left with the idea that pearl-necklace structures fluctuate significantly, although this tendency might be somewhat reduced for larger polymers, such as those with over a thousand monomers. This observation does not detract from the merits of the scaling pictures often developed for polymer research. The scaling pictures should not be taken too strictly as deterministic. They remain very useful in defining general trends and guide/interpret experiments.

Among the topics that we did not mention, dynamical aspects are likely the most important. A description of PE dynamics can be found in the review [40]. See also refs. [47,71,72] for collapse dynamics and ref. [73] on unfolding of polyelectrolyte condensates in electric fields. Recently, the occurrence of long-lived metastable knots in PAs has been investigated in relation with proteins [74].

The interaction of PEs with (biological) membranes and surfactants (phospholipids) in general is ubiquitous. See, for example, the less specifically biology-oriented review by Lindman [75]. Beyond the strict scope of PEs, there are a number of systems mainly brought along by biophysics which start to benefit from the knowledge of electrostatics accumulated in soft matter physics. Typically, additional physics enters the related theories besides electrostatics. Proteins are PEs and electrostatics is an important component of protein science [76]. Recently, the structure of viruses where electrostatic interactions play an important role received increasing attention. The review [77] describes recent advances in understanding viral structures. Biophysics puts forward questions which are sometimes raised in the surfactant community regarding short-distance electrostatics, typically close to an interface [42,78] or dense charged media [55]. Membraneless organelles are often seen as PE coacervates. Sometimes biophysics introduces brand new concepts into physics, such as active matter. More complex models for membraneless organelles involving concepts beyond passive matter were proposed recently [79]; for a review, see [80].

## Data Availability

Data are contained within the article and supplementary materials.

## Supplementary Materials

The following supporting information can be downloaded at: https://www.mdpi.com/article/10.3390/polym15234593/s1, Molecular dynamics simulation description, Rules for constructing simplexes [64,65,66,81,82].
