# Supplementary material for: Polyelectrolytes: From Seminal Works to the Influence of the Charge Sequence
Source: Polymers (Basel). 2023 Nov 30;15(23):4593. doi: 10.3390/polym15234593 (PMC10708673; doi:10.3390/polym15234593)
Supplement: Supplementary file 1 [file polymers-15-04593-s001.zip › polymers-2599780-supplementary.pdf]

Supplementary Materials

Polyelectrolytes: From Seminal Works to the Influence of the Charge Sequence

Nam-Kyung Lee [1], Min-Kyung Chae [2], Youngkyun Jung [3], Albert Johner [4,*] and Jean-Francois Joanny [5]

[1] Department of Physics and Astronomy, Sejong University, Seoul 05006, Republic of Korea; lee@sejong.ac.kr
[2] National Institute for Mathematical Sciences, Daejeon 34047, Republic of Korea; mkc@nims.re.kr
[3] Supercomputing Center, Korea Institute of Science and Technology Information, Daejeon 34141, Republic of Korea; yjung@kisti.re.kr
[4] Institut Charles Sadron CNRS-Unistra, 6 rue Boussingault, 67083 Strasbourg, France
[5] Institut Curie, Physique des cellules et Cancer, Collège de France Soft Matter and Biophysics Chair, 11 , PSL University, Place Marcelin-Berthelot, 75231 Paris, France; jean-francois.joanny@college-de-france.fr

* Correspondence: albert.johner@ics-cnrs.unistra.fr

Molecular dynamics simulation description

We consider single PE and single PA chains with quenched random charge sequences in weakly poor solvent condition. The electroneutrality is imposed by counterions compensating the net charge on the PE and PA backbones with no added salt.

Each chain is modeled as a bead-spring chain consisting of $N = 202$ beads with diameter σ where all end monomers and every third monomer can carry a charge. There are $N_s = 68$ sites which can bear charges. For PE, $N_s = 68$ sites can be either neutral with 0 charge or carry a positive unit (elementary) charge +1. All charge sequences are generated by unbiased Markovian processes. In uncorrelated PE, each charge site can either be charged (+1) or remain neutral (0) with equal probability, satisfying a global average of $\langle Q \rangle = N_s/2$. We sampled 50 independent sequences satisfying the net charge condition of $Q = 22$, 28, and 34.

We also considered charge sequences with charge-charge correlations along the chain. In general, Markovian statistics can be represented by a 2×2 transfer matrix $\mathcal{M}$ whose elements $\mathcal{M}_{ij}$ are the conditional probabilities $p_{i,j}$ that a site in state j is followed by a site in state i, the indices i, j being 0 for a neutral site and + for a charged site. The transfer matrix $\mathcal{M}$ propagates the state $\{\langle q \rangle, 1 - \langle q \rangle\}$ where $\langle q \rangle$ represents the average charge per site along the chain. The transfer matrix can be expressed with two parameters $\langle q \rangle$ and λ which characterizes the correlation along the chain.

$$\mathcal{M} = \begin{bmatrix} \langle q \rangle + \lambda(1 - \langle q \rangle) & \langle q \rangle(1 - \lambda) \\ (1 - \langle q \rangle)(1 - \lambda) & \langle 1 - q \rangle + \lambda \langle q \rangle \end{bmatrix}. \tag{S1}$$

The average length (in number of sites) of charged (neutral) blocks increase with λ as $\langle m_+ \rangle = \frac{1}{(1-\langle q \rangle)(1-\lambda)}$ ($\langle m_0 \rangle = \frac{1}{\langle q \rangle(1-\lambda)}$).

In the simple case where there is no correlation along the sequence, $\lambda = 0$, the average

block size is 2, and the transfer matrix is:

$$\mathcal{M}_{\text{uncorr}} = \begin{bmatrix} \frac{1}{2} & \frac{1}{2} \\ \frac{1}{2} & \frac{1}{2} \end{bmatrix}.$$

For blocky sequences we consider here, $\lambda = 1/2$, the average block length 4, and the transfer matrix is:

$$\mathcal{M}_{\text{blocky}} = \begin{bmatrix} \frac{3}{4} & \frac{1}{4} \\ \frac{1}{4} & \frac{3}{4} \end{bmatrix}.$$

For PA, we considered random uncorrelated sequences. Specifically, $N_s = 68$ sites are assigned either a positively charge (+1) or a negative charge(-1) with equal probability. The ensemble of sequences satisfy a global average of $\langle Q \rangle = 0$ but each sequence can have net charges. The difference between the number of positive and negative charges determines net charge of each sequence. We select $40 - 45$ independent sequences that satisfy net charge condition $Q = 16$, 20, and 24 for investigations.

All simulations are performed with periodic boundary conditions at concentration $c = 2.02 \times 10^{-4}\,\sigma^{-3}$.

In simulations for both PA and PE, the non-electrostatic interaction between two particles (e.g., monomer-monomer, monomer-counterion, counterion-counterion) are modelled by a truncated-shifted Lennard-Jones(LJ) potential: $U_{\text{LJ}}(r) = 4\,\epsilon_{\text{LJ}}[(\sigma/r)^{12} - (\sigma/r)^6 - (\sigma/r_c)^{12} + (\sigma/r_c)^6]$ for $r < r_c$ and 0 elsewhere. Here ϵ_{LJ} and σ represent the strength and range of the LJ potential, respectively; r denotes the center-to-center distance between two interacting particles. The cutoff distance r_c is set to $2.5\ \sigma$ for monomer–monomer, and $2^{1/6}\sigma$ for monomer–counterion and counterion–counterion interactions. In simulations of random PE, the value of the interaction parameter is set to $\epsilon_{\text{LJ}} = 0.7\ k_{\text{B}}T$. In simulations of PA, it is set to $\epsilon_{\text{LJ}} = 0.6\ k_{\text{B}}T$ so that polymers are in moderately poor solvent condition. The chain connectivity is ensured by the finite extension nonlinear elastic (FENE) potential between two consecutive beads, $U_{\text{FENE}}(r) = -0.5kr_0^2 \ln[1 - (r/r_0)^2]$, where the spring constant is taken

as $k = 30\ k_{\rm B}T/\sigma^2$ and the maximum bond length as $r_0 = 1.5\ \sigma$. A pair of charged particles i and j, located at $\vec{r}_i$ and $\vec{r}_j$, interact via the Coulomb potential $U_{\rm C}(r_{ij}) = k_{\rm B}Tl_{\rm B}/r_{ij}$. The strength of the electrostatic interactions is determined by the Bjerrum length $l_{\rm B}$. In both simulations it is set to $l_{\rm B} = 3\sigma$. The long-range electrostatic interactions are calculated by the particle-particle-particle-mesh (PPPM) method implemented in LAMMPS software package.[1]

In order to explore the phase space, we integrated Newton's equations of motion using the velocity Verlet algorithm with an integration time step $\delta t = 0.01\ \tau$, where $\tau = \sigma(m/k_{\rm B}T)^{1/2}$ is the characteristic time scale with bead mass $m = 1$. A Langevin thermostat with the damping constant $1.0\ \tau^{-1}$ was used to keep the system at the fixed temperature $k_{\rm B}T = 1.0$.

In simulations of PE, we first performed 4×10^6 integration steps (which is equivalent to $4 \times 10^6 \delta t = 4 \times 10^6 \times 0.01\tau = 4 \times 10^4\tau$) in order for the mean square radius of gyration of the chain to relax to their equilibrium values. After equilibration, we ran additional 1.2×10^7 ($= 1.2 \times 10^5\tau$) integration steps and collected data every 5×10^3 ($= 50\tau$) time steps. In simulations in PA, after $6 \times 10^4\tau$ equilibration times, we simulated for $4 \times 10^5\tau$ and collect data every 10τ.

To identify pearl-necklace structures, we first identify monomers that belong to a globular pearl or to a string. We used the algorithm suggested by Liao *et al*[2] as in our previous studies.[3–5] First, we make lists of monomers belonging to globules such that the number of neighboring monomers within the sphere of cutoff radius $R_{\rm cut} = 2.0\,\sigma$ is larger than $N_{cut} = 10$ in simulations of PE (while, in PA simulations, we used $R_{\rm cut} = 2.3\,\sigma$ and $N_{\rm cut} = 8$). This choice of $R_{\rm cut}$ is justified by the pair correlation function discussed in our previous studies.[3–5] Other monomers are considered as parts of strings. In second step, we eliminate loops that begin and end at the same globule. These procedures are repeated until the lists no longer change.

Rule for constructing Simplexes

We consider a state of $n = n_l + n_s$ pearls comprising of n_l large pearls and n_s small pearls. The set of asymmetry parameters for the mass distribution $\{x_i\}$ are defined by $m_i/M = 1/n + x_i$ for pearls with mass distribution of $\{m_i\}$ with M being total mass in pearls. We rank the pearls by decreasing mass (by decreasing x_i), which also avoids multiple counting of states. An n-pearl state can be represented by $(n-1)$ mass asymmetry values x_i, $i = 1, 2, ...n-1$ with constraint $\Sigma_{i=1}^{i=n} x_i = 0$. The interior of the simplex is defined by the following n inequalities.

- $x_i \geq x_{i+1}$ for $1 \leq i \leq n-2$,
- $x_{n-1} \geq x_n = -\sum_{i=1}^{n-1} x_i$,
- $x_n = -\sum_{i=1}^{n-1} x_i \geq -1/n$.

The first $n-1$ conditions pertain to ranking conditions of pearl mass and the last condition ensures that the smallest n-th pearl has a positive mass. For two pearls, the points are bound to the segment (1-simplex). In the case of three pearls, the representative points are bound to a triangle (2-simplex) and in the case of four beads to a tetrahedron (3-simplex).

There are n vertices $V_{n,0}$, $V_{n-1,1}$, $V_{n-2,2}$, ..., $V_{1,n-1}$ in $(n-1)$-simplex. In notation V_{n_l,n_s}, the first index in the subscript refers to the number of large pearls (n_l) and the second index to the number of small pearls (n_s). Explicitly, the coordinates of vertices can be obtained as $\{\frac{n_s}{n_l n}, ..., -\frac{1}{n}, ...\}$, where the first value repeats n_l times and the second n_s times with (one of) the smallest value(s) omited. Table S1 provides a summary of the vertex locations for $(n-1)$ simplex.

Table S1: The positions of the vertices of $n-1$ simplex for $n = 2$, 3, and 4.

	$V_{n,0}$	$V_{n-1,1}$	$V_{n-2,2}$	$V_{n-3,3}$
$n = 2$	0	1/2	-	-
$n = 3$	(0, 0)	(1/6, 1/6)	(2/3, -1/3)	-
$n = 4$	(0, 0, 0)	(1/12, 1/12, 1/12)	(1/4, 1/4, -1/4)	(3/4, -1/4, -1/4)

The position of energy extrema E_{n_l,n_s} ($E_{n,0}$, $E_{n-1,1}$, $E_{n-2,2}$, ..., $E_{1,n-1}$) in the $(n-1)$ simplex can be written as $\{x_l, ..., x_s, ...\}$, where the first value repeats n_l times and the second n_s times. With given n_l and n_s, the n_l (n_s) large (small) pearls have mass asymmetry value of x_l (x_s). Only x_l is a independent variable and $x_s = -\frac{n_l}{n_s}x_l$. The mass asymmetry values x_l of large pearls are expressed using the parameter u:

$$x_l = \frac{1}{n_l + n_s}\frac{1-u^3}{\frac{n_l}{n_s} + u^3}, \tag{S2}$$

and reduce to a polynomial equation in u with a trivial solution u =1 corresponding to the symmetric $\vec{0}$ state (i.e. evenly sized pearls). Other extrema satisfy the quartic equation:

$$0 = \frac{\chi n_s}{2}u^4 + (\frac{\chi}{2} - n_s)n_s u^3 - 2n_l n_s u^2 + (\frac{\chi}{2} - n_l)n_l u + \frac{\chi}{2}n_l. \tag{S3}$$

For $\chi < n$, there are two real solutions. A solution corresponding to a positive value of x_l characterizes a large pearl. A negative value characterizes the small pearl of the state with n_l small pearls and n_s large pearls. Table S2 show the location $x_l = x_1$ for energy extrema E_{n_l,n_s} evaluated for $\chi = 1.5$ using Eqs. S1 and S2. Note that in the limit of vanishing χ, E_{n_l,n_s} merge to V_{n_l,n_s}. In the opposite limit where χ approaches to n, the energy of extrema become closer. See, for example, $n = 2$ case in Table S2.

Table S2: The energy values at energy extrema E_{n_l,n_s} and their coordinates in $(n-1)$ simplexes. The energy and mass asymmetry values are obtained for $\chi = 1.5$. The lowest energy state is denoted by the color red.

	$E_{n,0}$	$E_{n-1,1}$	$E_{n-2,2}$	$E_{n-3,3}$
$n = 2$	-0.295	-0.306	-	-
	(0)	(0.3467)	-	-
$n = 3$	-0.337	-0.372	-0.415	-
	(0,0)	(0.154, 0.154)	(0.573, -0.286)	-
$n = 4$	-0.317	-0.366	-0.418	-0.484
	(0,0,0)	(0.081, 0.081, 0.081)	(0.237, 0.237, -0.237)	(0.679,-0.226,-0.226)

References

(1) Plimpton S. Fast parallel algorithms for short-range molecular dynamics. *J. Comput. Phys.* **1995**, *117*, 1-19. Also see http://lammps.sandia.gov.

(2) Liao, Q.; Dobrynin, A. V.; Rubinstein, M. Counterion-correlation-induced attraction and necklace formation in polyelectrolyte solutions: Theory and simulations. *Macromolecules* **2006**, *39*, 1920-1938.

(3) Chae, M. -K.; Lee, N. -K.; Jung, Y.; Johner, A.; Joanny, J. F. Partially globular conformations from random charge sequences. *ACS Macro Lett.* **2022**, *11*, 382-386.

(4) Chae, M. -K.; Lee, N. -K.; Jung, Y.; Johner, A.; Joanny, J. F. Structure of a hydrophobic polyelectrolyte chain with a random sequence. *Macromolecules* **2022**, *55*, 6275-6285.

(5) Chae, M. -K.; Lee, N.-K.; Jung, Y.; Johner, A. Shape fluctuations of random polyampholyte and intrinsically disordered protein sequences. *Macromolecules*, **2023**, *56*, 785-793.
